# TAU-INDUCED ASTROCYTE SENESCENCE: A NOVEL MECHANISM FOR NEURONAL DYSFUNCTION IN ALZHEIMER’S DISEASE

**DOI:** 10.1093/geroni/igz038.348

**Published:** 2019-11-08

**Authors:** Angela Olson, Stacy A Hussong, Rakez Kayed, Veronica Galvan

**Affiliations:** 1 Barshop Institute for Longevity and Aging Studies, San Antonio, Texas, United States; 2 University of Texas Health Science Center at San Antonio, San Antonio, Texas, United States; 3 Dept. of Neurology, University of Texas Medical Branch at Galveston, Galveston, Texas, United States

## Abstract

Chronic sterile inflammation is a pathological feature of Alzheimer’s disease (AD) and other neurodegenerative diseases. The mechanisms that drive neuroinflammation and its impact on AD progression are still incompletely understood. The accumulation of molecular damage in somatic cells can trigger cellular senescence, an irreversible state of cell cycle arrest accompanied by the expression of proinflammatory mediators known collectively as the “senescence-associated secretory phenotype” (SASP). Misfolded tau forms pathogenic soluble aggregates that are released extracellularly and are transmitted trans-neuronally, promoting native tau phosphorylation and aggregation in target cells. We recently showed that, in addition to neurons, pathogenic soluble extracellular tau aggregates propagate to brain microvascular endothelial cells, where microtubule destabilization triggers senescence/SASP. Because astrocytes have a critical role in the regulation of both synaptic function and cerebral blood flow and are directly exposed to tau at its site of release at the tripartite synapse, we conducted studies to define whether soluble aggregated tau propagates to astrocytes, inducing astrocyte senescence/SASP and neuronal dysfunction/damage. Our studies indicate that tau can be propagated transcellularly to astrocytes, triggering cellular senescence/SASP. Our studies suggest that astrocyte senescence is detrimental to dendritic and synaptic structure and density, suggesting that pathogenic soluble tau-induced astrocyte senescence may contribute to synaptic dysfunction and loss in AD. Drugs that eliminate senescent cells are FDA-approved and antibody-based approaches to remove tau from brain are already in clinical trials. Our studies suggest that these interventions could be effective in the treatment of AD and other tauopathies.

